# Risk Factors, Molecular Epidemiology and Outcomes of Ertapenem-Resistant, Carbapenem-Susceptible *Enterobacteriaceae*: A Case-Case-Control Study

**DOI:** 10.1371/journal.pone.0034254

**Published:** 2012-03-26

**Authors:** Jocelyn Teo, Yiying Cai, Sarah Tang, Winnie Lee, Thean Yen Tan, Thuan Tong Tan, Andrea Lay-Hoon Kwa

**Affiliations:** 1 Department of Pharmacy, Singapore General Hospital, Singapore, Singapore; 2 Department of Laboratory Medicine, Changi General Hospital, Singapore, Singapore; 3 Department of Infectious Diseases, Singapore General Hospital, Singapore, Singapore; Los Angeles Biomedical Research Institute, United States of America

## Abstract

**Background:**

Increasing prevalence of ertapenem-resistant, carbapenem-susceptible *Enterobacteriaceae* (ERE) in Singapore presents a major therapeutic problem. Our objective was to determine risk factors associated with the acquisition of ERE in hospitalized patients; to assess associated patient outcomes; and to describe the molecular characteristics of ERE.

**Methods:**

A retrospective case-case-control study was conducted in 2009 at a tertiary care hospital. Hospitalized patients with ERE and those with ertapenem-sensitive *Enterobacteriaceae* (ESE) were compared with a common control group consisting of patients with no prior gram-negative infections. Risk factors analyzed included demographics; co-morbidities; instrumentation and antibiotic exposures. Two parallel multivariate logistic regression models were performed to identify independent variables associated with ERE and ESE acquisition respectively. Clinical outcomes were compared between ERE and ESE patients.

**Results:**

Twenty-nine ERE cases, 29 ESE cases and 87 controls were analyzed. Multivariate logistic regression showed that previous hospitalization (Odds ratio [OR], 10.40; 95% confidence interval [CI], 2.19–49.20) and duration of fluoroquinolones exposure (OR, 1.18 per day increase; 95% CI, 1.05–1.34) were unique independent predictors for acquiring ERE. Duration of 4^th^-generation cephalosporin exposure was found to predict for ESE acquisition (OR, 1.63 per day increase; 95% CI, 1.05–2.54). In-hospital mortality rates and clinical response rates were significantly different between ERE and ESE groups, however ERE infection was not a predictor of mortality. ERE isolates were clonally distinct. Ertapenem resistance was likely to be mediated by the presence of extended-spectrum β-lactamases or plasmid-borne AmpC in combination with impermeability due to porin loss and/or efflux pumps.

**Conclusion:**

Prior hospitalization and duration of fluoroquinolone treatment were predictors of ERE acquisition. ERE infections were associated with higher mortality rates and poorer clinical response rates when compared to ESE infections.

## Introduction

Extended-spectrum β-lactamases (ESBL)-producing *Enterobacteriaceae* are major contributors to the mounting gram-negative resistance problem globally [1]. Carbapenems are often the only remaining therapeutic options available for these serious ESBL-producing *Enterobacteriaceae* infections [2]. Unfortunately, carbapenem resistance has also emerged as a result of selective pressure on *Enterobacteriaceae* [3]. Whilst Singapore is not spared from this emergence, it is fortunate that overall rates of susceptibility to carbapenems remained fairly stable [4]. However, since 2006, systemic surveillance of hospitals unexpectedly detected an increasing prevalence of *Klebsiella pneumoniae* and *Escherichia coli* clinical isolates that were resistant to ertapenem, but susceptible to group II carbapenems [5]. This coincided with the increasing popularity of ertapenem as a treatment option for ESBL- and AmpC-producing *Enterobacteriaceae* infections, in spite of the lack of prospective comparative trials investigating its use in such infections.

Carbapnem resistance may be mediated by carbapenemases or metallo-β-lactamases [6]; or due to a combination of ESBL or AmpC β-lactamase production with impermeability caused by porin loss [7]–[10]. *Enterobacteriaceae* exhibiting low-level resistance to ertapenem, a Group I carbapenem, may still remain susceptible to Group II carbapenems such as imipenem and meropenem. The underlying resistance mechanisms in these unique ertapenem-resistant *Enterobacteriaceae* appear to differ from those isolates with universal resistance to all carbapenems – ertapenem permeability tends to be more affected by porin loss compared to other Group II carbapenems and ertapenem resistance is seldom carbapenemases-mediated [7], [11], [12].

The clinical epidemiology of these ertapenem-resistant, Group II carbapenem-susceptible *Enterobacteriaceae* (ERE) is poorly understood. There have been several reports regarding the risk factors for acquisition of carbapenem-resistant *Enterobacteriaceae* [13]–[19], but less so for ertapenem resistance in particular [20], [21]. The intent of this study was to identify the risk factors associated with the acquisition of ERE among hospitalized patients, to compare the outcomes of patients with ERE with those with ertapenem-susceptible *Enterobacteriaceae* (ESE) infections, and to describe the molecular profile of ERE locally. Identification of factors contributing to the emergence of ERE among the hospitalized population is crucial in the design of effective strategies to prevent the development of such infections, as well as to avoid therapy failure by precluding empiric ertapenem use in this risk population.

## Materials and Methods

### Study setting

The study was conducted at Singapore General Hospital, a 1,600-bedded acute tertiary care hospital between January 2009 to September 2009. The hospital is the largest of six public healthcare hospitals, and accounted for approximately 35% of recorded inpatient-days among them. Infection control policies remained unchanged during the study period. ERE prevalence remained stable and there was no indication of an outbreak during this period. Surveillance cultures to detect asymptomatic ERE colonization were not routinely performed in the institution. The study was reviewed and approved by the Singhealth institutional ethics review board. As this was a retrospective study, the need for an informed consent was waived by the ethics review board.

### Study design

A retrospective parallel case-control study design was used to identify risk factors associated with the acquisition of ERE. The case-case-control design overcomes several limitations of the standard case-control design, particularly studies where patients infected with “susceptible phenotype” are elected as controls. In such case-control studies, effect measures of antibiotics active against the susceptible, but not the resistant phenotype, could be falsely inflated since the administration of these antibiotics close to the time of culture would have prevented the recovery of the susceptible phenotype, hence excluding these patients from the control group. The other advantages of the case-case-control methodology are described in detail elsewhere [22], [23].

The computerized records of the hospital's clinical microbiological laboratory were used to identify all clinical cultures positive for *Enterobactaeriaceae*. The first case group consisted of all adult inpatients (>18 years old) with microbiological cultures positive for ERE, regardless of whether the culture reflected a clinically-relevant infection or colonization. The second case group consisted of adult inpatients with microbiological cultures positive for ESE recorded within the same study period. In order to ensure that the two parallel case-control models were comparable, an equal number of (i) *Enterobacteriaceae* species type and (ii) specimen type were selected for each case group. Patients who had multiple cultures positive for *Enterobacteriaceae* were included only once and the “index” culture is the first *Enterobacteriaceae* specimen recorded during the stay that qualified each patient for inclusion into the two case groups.

Control group patients were adult inpatients admitted within the study period with no history of prior infection with *Enterobacteriaceae* or other gram-negative bacilli, but could have gram-positive infections. Three control patients were randomly identified and chosen from the hospital's computerized administrative source records for each ERE/ESE case patient.

An independent observer (a research assistant), blinded to the study's hypothesis/objectives, performed random selection of ESE case and control patients based solely on the respective inclusion and exclusion criteria provided to him. The first patient listed in the database who met the study criteria was selected and this process was continued until all ESE cases and controls patients were identified.

### Data collection

Data were sought from inpatient charts, electronic medical records, clinical microbiology laboratory computerized databases and collated in a structured data collection form by three trained reviewers. Variables analyzed as risk factors included: 1) demographics (age, gender); 2) presence of comorbid conditions and Charlson weighted co-morbidity index; 3) severity of illness as determined by APACHE II score; 4) hospitalization history such as previous hospital and nursing home stay, previous ICU stay, length of hospital stay prior to outcome of interest (defined as length of stay prior to *Enterobacteriaceae* isolation for case patients and total length of stay for controls); 5) exposure to invasive interventions (central lines, urinary catheters, drainage devices, invasive ventilation, dialysis, non-surgical invasive procedures such as endoscopic procedures, invasive surgery, dialysis); 6) receipt of immunosuppressive therapy (defined as receipt of >one dose of chemotherapy or immunosuppressants, or >14 days of corticosteroids at an equivalent daily dose of 20 mg prednisolone) and 7) antibiotics exposure (receipt of ≥one dose of various classes of antimicrobials). The number of invasive devices and cumulative duration of antibiotics were also tabulated. All variables were evaluated for an interval of 90 days prior to the occurrence of ERE/ESE for case patients or end of hospitalization for control patients.

A retrospective cohort study involving the same groups of ERE and ESE patients were conducted in order to evaluate the impact of ERE on clinical outcomes. All ERE and ESE patients who were considered having clinically relevant infections were included for outcome analyses. Infection was considered clinically relevant if *Enterobacteriaceae* were isolated from clinical specimens obtained from sterile sites (*e.g.* blood or pleural effusion); or if there was a presence of clinical symptoms or signs of infection consistent with the infecting *Enterobacteriaceae*; or antibiotic therapy was administered for the *Enterobacteriaceae* infection. Colonizers were excluded. For polymicrobial infections, patients were included only if the presence of ERE or ESE contributed to the decision for antibiotic treatment. Primary outcomes of interest were: (i) the final end-points of hospitalization *i.e.* discharge or mortality (in-hospital all-cause mortality), (ii) hospital length of stay after infection, (iii) 30-day readmission (defined as non-elective readmission within 30 days of discharge), and (iv) clinical response (defervescence, resolution of abnormalities of vital signs and infection markers, or resolution of symptoms specific to infection). Clinical improvement was presumed if patient was well enough to be discharged in patients where surrogate infection markers were not documented.

### Microbiological methods

Carbapenem susceptibility was determined using disk diffusion and interpreted in accordance to the 2009 Clinical and Laboratory Standards Institute guidelines as per hospital's clinical microbiology laboratory protocol [24]. Susceptibility testing for ertapenem and imipenem was part of laboratory's routine protocol, but not for meropenem. *Enterobacteriaceae* isolates with “intermediate” or “resistant” classification for ertapenem, but “susceptible” to class II carbapenems, were included in ERE case group. Fifteen clinical isolates were available for further molecular characterization. A conventional rep-polymerase chain reaction (PCR) method was used for clonal typing [25]. PCR products were analyzed by chip-based microfluidic electrophoresis (Experion, Biorad, USA). Digitalized banding images were exported and cluster analysis was performed using Bionumerics 5.4 (AppliedMaths, Kortrijk, Belgium). The isolates were considered as indistinguishable (similarity of DNA fragment pattern >90%) or distinct (similarity of DNA fragment pattern <90%). Resistance gene analyses of ESBLs, plasmid-mediated AmpC genes, metallo-β-lactamases (MBLs) and *Klebsiella pneumoniae* carbapenemase (KPC) were performed using modified methods from previously published PCR protocols [26]–[30].

### Statistical Analysis

Catergorical variables were presented as numbers and percentages, and were compared using the Χ^2^ or Fisher exact test, as appropriate. Continuous variables were presented as mean ± SD or median and range, and were compared using the Student's t test or the Mann-Whitney test, depending on the validity of the normality assumption.

In the risk factor analysis, multivariate logistic regression models were used to compare each case group to control group. Clinically plausible variables identified in the univariate analysis were included in a stepwise selection multivariate logistic regression model if P<0.1. The final model was chosen on basis of biologic plausibility. Odds ratios (OR) and 95% confidence intervals (CI) were calculated to evaluate the strength of any association. Results from the ERE-control model were compared with the ESE-control model to identify significant factors unique to each case group. In the outcomes analysis, ERE and ESE patients who died were compared with those who survived in order to determine risk factors predicting for in-hospital mortality via univariate and subsequently multivariate logistic regression analysis.

For all calculations, a 2-tailed P value of less than 0.05 was considered to reveal a statistical significant difference. Statistical analyses were performed using SPSS 17.0 (SPSS, Chicago, IL, USA).

## Results

### Study population

During the study period, 29 patients with ERE, 29 patients with ESE and 87 controls were identified. Of the ERE species included in the study, *Klebsiella spp.* (16/29, 55.2%) was most commonly identified, while *Escherichia coli* (5/29, 17.2%) and *Enterobacter spp.* (8/29, 27.6%) accounted for the remaining ERE species. Urine (12/29, 41.4%) was the most common site which ERE were isolated from, followed by blood (6/29, 20.7%), abdominal tissues/fluids (6/29, 20.7%), respiratory secretions (3/29, 10.3%) and skin and soft tissue wounds (2/29, 6.9%) (Table 1). Of the 29 ERE isolates, 20 (69.0%) were resistant to ertapenem and 9 (31.0%) were intermediate. These ERE isolates exhibited multi-drug resistance – 100% was resistant to ceftriaxone and amoxicillin-clavulanate; 97% to piperacillin-tazobactam; 79% to ciprofloxacin; and 76% to cefepime. However, ERE remained relatively susceptible to amikacin (3% resistant) and gentamicin (41% resistant).

**Table 1:** Species, site and resistance mechanisms of ERE isolates.

	Species		
	*E.coli*	*Klebsiella spp.*	*Enterobacter spp.*
Site	n = 5	n = 16	n = 8
Blood	4 (13.8)	0	2 (6.9)
Urine	1 (3.4)	10 (34.5)	1 (3.4)
Respiratory	0	2 (6.9)	1 (3.4)
Skin/soft tissue	0	0	2 (6.9)
Abdominal	0	4 (13.8)	2 (6.9)
**Resistance mechanisms**			
*Isolates tested*	*1 (20.0)*	*9 (56.3)*	*5 (62.5)*
SHV	0	8 (88.9)	1 (20.0)
TEM	1 (100.0)	7 (77.8)	1 (20.0)
CTX-M	1 (100.0)	7 (77.8)	1 (20.0)
AmpC	0	6 (66.7)	0
KPC	0	0	0
Metallo-β-lactamase	0	0	0

Data are presented as n(%), unless otherwise stated.

The demographic characteristics and co-morbidities of the case and control patients are shown in Table 2. Overall, ERE and ESE case patients were fairly similar in baseline characteristics to the control group respectively. However, ERE patients were more likely to have cardiovascular and hepatic diseases, while ESE patients were older, more likely to have hepatic and renal diseases and had a higher Charlson co-morbidity index, when compared to the control group.

**Table 2:** Baseline demographic characteristics and co-morbidities.

	ERE	ESE	Control	ERE versus controls		ESE versus controls	
	(n = 29)	(n = 29)	(n = 87)	OR (95% CI)	*P*	OR (95% CI)	*P*
Median age, yr (range)	55 (22–91)	75 (27–88)	65 (18–100)	…	0.22	…	0.01
Male sex	12 (41.4)	15 (51.7)	36 (41.3)	1.15 (0.49–2.69)	0.75	1.52 (0.65–3.53)	0.33
Diabetes mellitus	11 (37.9)	15 (51.7)	32 (36.8)	1.05 (0.44–2.50)	0.91	1.84 (0.79–4.30)	0.16
Cardiovascular disease	15 (51.7)	8 (27.6)	25 (28.7)	3.05 (1.28–7.26)	0.01	0.95 (0.37–2.41)	0.91
Hepatic disease	4 (13.8)	5 (17.2)	4 (4.6)	4.32 (1.08–17.37)	0.04	4.32 (1.08–17.37)	0.04
Renal disease	9 (31.0)	12 (41.4)	19 (21.8)	1.61 (0.63–4.11)	0.32	2.53 (1.03–6.20)	0.04
Neurologic disease	6 (20.7)	5 (17.2)	11 (12.6)	1.80 (0.60–5.41)	0.36	1.44 (0.46–4.56)	0.54
Malignancy	8 (27.6)	5 (17.2)	15 (17.2)	1.77 (0.68–4.90)	0.23	1.00 (0.33–3.04)	1.00
Median Charlson score (range)^a^	4 (0–13)	6 (0–10)	4 (0–13)	…	0.09	…	<0.001
Median APACHE II score, (range)^a^	15 (2–30)	15 (2–32)	9 (0–32)	…	0.06	…	0.005

The ERE-control model was conducted independently from the ESE-control model.

Data are presented as n(%), unless otherwise stated.

a Measured on date of culture isolation for case patients and on date of admission for controls.

Comparing ERE cases to controls, ERE patients were significantly more likely to have been previously exposed to the hospital environment and various instrumentation or interventions, and had longer hospital stays prior outcome of interest (Table 3). Likewise ESE patients had longer hospital stays prior outcome of interest when compared to controls. However, unlike ERE cases, only exposure to central lines and urinary catheters were significantly different between the ESE and control groups.

**Table 3:** Hospital exposures and instrumentation/interventions as risk factors for ERE infections.

	ERE	ESE	Control	ERE versus controls		ESE versus controls	
	(n = 29)	(n = 29)	(n = 87)	OR (95% CI)	*P*	OR (95% CI)	*P*
Immunosuppression	7 (24.1)	3 (10.3)	4 (4.6)	6.60 (1.77–24.60)	0.005	2.39 (0.50–11.40)	0.36
Previous hospital stay	23 (79.3)	15 (51.7)	29 (33.3)	7.67 (2.81–20.90)	<0.001	2.14 (0.91–5.03)	0.08
Median length of stay prior to outcome of interest^a^, days(range)	17 (1–85)	3 (1–47)	6 (1–40)	…	0.004	…	0.71
Central lines	18 (62.1)	9 (28.1)	8 (9.2)	16.20 (5.69–45.93)	<0.001	4.44 (1.52–12.97)	0.01
Median no of central lines, (range)	1 (0–3)	0 (0–1)	0 (0–2)	…	<0.001	…	0.07
Urinary catheter	18 (62.1)	15 (51.7)	18 (20.7)	5.43 (2.20–13.40)	<0.001	4.11 (1.68–10.04)	0.001
Nasogastric tube	11 (37.9)	6 (18.8)	8 (9.2)	5.20 (1.81–14.94)	0.002	2.58 (0.81–8.18)	0.11
Drainage device	13 (44.8)	5 (15.6)	7 (8.0)	8.07 (2.77–23.50)	<0.001	2.38 (0.69–8.19)	0.17
Median no of drainage devices, (range)	0 (0–5)	0 (0–3)	0 (0–3)	…	0.01	…	0.16
Surgery	12 (41.4)	8 (25.0)	20 (23.0)	2.37 (0.97–5.77)	0.06	1.28 (0.49–3.32)	0.62
Non-surgical invasive procedure	19 (65.5)	12 (37.5)	26 (29.9)	4.46 (1.83–10.89)	0.001	1.66 (0.69–3.95)	0.25
Invasive ventilation	6 (20.7)	3 (9.4)	3 (3.4)	5.83 (1.30–26.18)	0.02	3.23 (0.62–16.99)	0.16

The ERE-control model was conducted independently from the ESE-control model.

Data are presented as n(%), unless otherwise stated.

a Refers to duration of hospital stay prior to culture isolation for cases and entire duration of hospital stay for controls.

More than 70% of patients in each group received some form of antibiotic in the 90 days prior to the outcome of interest (Table 4). ERE patients received significantly longer durations of all classes of antibiotics investigated, with the exception of penicillin, when compared to controls. In contrast, there were no significant differences in antibiotic exposures between ESE patients and controls, with the exception of the receipt of 4^th^-generation cephalosporins.

**Table 4:** Antibiotic exposures^a^ as risk factors for ERE infections.

	ERE	ESE	Control	ERE versus controls		ESE versus controls	
	(n = 29)	(n = 29)	(n = 87)	OR (95% CI)	*P*	OR (95% CI)	*P*
Any antibiotic	28 (96.6)	24 (82.8)	62 (71.3)	11.29 (1.45–87.53)	0.005	1.94 (0.66–5.64)	0.22
Penicillins	7 (24.1)	5 (17.2)	13 (14.9)	1.81 (0.64–5.10)	0.26	1.19 (0.38–3.67)	0.77
Penicillins duration	0 (0–37)	0 (0–11)	0 (0–10)	…	0.11	…	0.83
Beta-lactam/beta-lactamase inhibitors	23 (79.3)	12 (41.4)	41 (47.1)	5.39 (1.88–15.41)	0.001	0.79 (0.34–1.85)	0.29
Beta-lactam/beta-lactamase inhibitors duration	7 (0–30)	0 (0–23)	0 (0–31)	…	0.004	…	0.96
3^rd^-generation cephalosporins	18 (62.1)	14 (48.3)	27 (31.0)	3.64 (1.51–8.74)	0.003	2.07 (0.88–4.89)	0.09
3^rd^-generation cephalosporins duration	3 (0–14)	0 (0–10)	0 (0–15)	…	0.01	…	0.25
4^th^-generation cephalosporins	7 (24.1)	4 (13.8)	1 (1.1)	32.76 (3.88–276.49)	<0.001	13.76 (1.47–128.75)	0.01
4^th^-generation cephalosporins duration	0 (0–11)	0 (0–17)	0 (0–6)	…	0.02	…	0.08
Ertapenem	3 (10.3)	1 (3.1)	0	…	0.01	…	0.25
Ertapenem duration	0 (0–25)	0 (0–9)	0	…	0.17	…	0.33
Anti-pseudomonal carbapenems	6 (20.7)	2 (6.9)	3 (3.4)	7.30 (1.70–31.47)	0.01	2.07 (0.33–13.07)	0.60
Anti-pseudomonal carbapenems duration	0 (0–28)	0 (0–7)	0 (0–12)	…	0.07	…	0.47
Fluoroquinolones	12 (41.4)	5 (17.2)	17 (19.5)	2.91 (1.17–7.22)	0.02	0.86 (0.29–2.58)	0.78
Fluoroquinolones duration	0 (0–90)	0 (0–19)	0 (0–18)	…	0.01	…	0.20
Aminoglycosides	8 (27.6)	1 (3.4)	4 (4.6)	7.91 (2.17–28.78)	0.002	0.74 (0.08–6.91)	1.00
Aminoglycosides duration	0 (0–12)	0 (0–6)	0 (0–5)	…	0.02	…	0.60

The ERE-control model was conducted independently from the ESE-control model.

Categorical data are presented as n (%), while continuous data are presented as median days (range).

a Defined as receipt of ≥one dose of antimicrobials 90 days prior to *Enterobacteriaceae* isolation for case patients or end of hospitalization for control patients.

### Risk factor analysis

The results for the multivariate analyses of the ERE-control and ESE-control models are presented in Table 5. When the two models were contrasted, previous hospital stay and longer exposures of fluoroquinolone use were unique significant predictors of ERE acquisition, while duration of exposure to 4^th^-generation cephalosporins was the only unique significant predictor of ESE acquisition.

**Table 5:** Multivariate model of risk factors for *Enterobacteriaceae* acquisition.

	ERE versus controls		ESE versus controls	
	OR (95% CI)	*P*	OR (95% CI)	*P*
Previous hospital facility stay	10.40 (2.19–49.20)	0.003		
Urinary catheter	9.70 (2.33–40.37)	0.002	3.33 (1.24–9.00)	0.02
Median duration of fluoroquinolone, days (range)	1.18 (1.05–1.34)^a^	0.007		
Median duration of 4^th^-generation cephalosporin, days (range)			1.63 (1.05–2.54)^a^	0.03

The ERE-control model was conducted independently from the ESE-control model. Other covariates not presented had a *P* value>0.05.

a OR corresponds to a unit increase in the continuous scale of the variable.

### Outcomes Analysis

Twenty-six patients with ERE and 27 patients with ESE were considered to have clinically-relevant *Enterobacteriaceae* infections and were receiving antibiotic therapy for these infections. In-hospital mortality was significantly higher in the ERE group (P = 0.04) (Table 6). ERE patients also had a poorer clinical response rate (P = 0.04).

**Table 6:** Outcomes for ERE and ESE infections.

Outcomes	ERE (n = 26)	ESE (n = 27)	*P*
In-hospital mortality	8 (30.8)	2 (7.4)	0.04
Median hospital days after infection, (range)	16 (1–107)	12 (2–163)	0.39
30-day readmission^a^	7 (38.9)	9 (36.0)	0.30
Clinical response	17 (65.4)	24 (88.8)	0.04

a Analyzed as percentage of patients who were discharged.

Table 7 showed the results of univariate analysis for risk factors associated with in-hospital mortality among the ERE and ESE-infected patients. In-hospital mortality was associated with malignancy, high APACHE II score, isolation of *Enterobacteriaceae* in blood and ERE infection. However, none of these variables remained significant when introduced into the multivariate model.

**Table 7:** Univariate predictors of in-hospital mortality among ERE and ESE patients.

	Died (n = 10)	Survived (n = 43)	OR (95% CI)	*P*
Median age, yr (range)	62 (22–91)	69 (24–88)	…	0.31
Male sex	7 (70.0)	19 (44.2)	1.94 (0.67–12.95)	0.18
Diabetes mellitus	6 (60.0)	17 (39.5)	2.29 (0.56–9.35)	0.30
Cardiovascular disease	5 (50.0)	17 (39.5)	1.53 (0.38–6.09)	0.72
Hepatic disease	2 (20.0)	7 (16.3)	1.29 (0.22–7.39)	1.00
Renal disease	2 (20.0)	17 (39.5)	0.38 (0.07–2.02)	0.30
Neurologic disease	2 (20.0)	8 (18.6)	1.09 (0.19–6.17)	1.00
Malignancy	5 (50.0)	6 (14.0)	6.17 (1.36–27.92)	0.02
Median Charlson score (range)^a^	5 (2–13)	5 (0–12)	…	0.90
Median APACHE II score, (range)^a^	19 (12–30)	15 (2–46)	…	0.05
Previous hospital stay	8 (80.0)	28 (65.1)	2.14 (0.40–11.40)	0.47
Previous ICU stay	2 (20.0)	7 (16.3)	1.28 (0.22–7.39)	1.00
Immunosuppression	3 (30.0)	6 (14.0)	2.64 (0.53–13.15)	0.35
**Specimen type**				
*Blood*	5 (50.0)	7 (16.3)	5.14 (1.17–22.61)	0.04
*Urine*	2 (20.0)	18 (41.9)	0.35 (0.07–1.83)	0.29
*Respiratory secretions*	1 (10.0)	5 (11.6)	0.84 (0.09–8.15)	1.00
*Skin/ Soft tissue*	0	4 (9.3)	…	1.00
*Abdominal*	2 (20.0)	9 (20.9)	0.94 (0.17–5.25)	1.00
ERE infection	8 (80.0)	18 (41.9)	5.55 (1.05–29.33)	0.04
Appropriate therapy^b^	4 (40.0)	7 (16.3)	3.43 (0.76–15.40)	0.19

Data are presented as n(%), unless otherwise stated.

a Measured on date of culture isolation for case patients and on date of admission for controls.

b Defined as receipt of an antibiotic which *Enterobacteriaceae* was susceptible to within 24 hours of culture.

### Molecular characteristics

Fifteen isolates (15/29, 51.7%) were available for further molecular work-up, which included nine *K. pneumoniae*, four *E. cloacae*, one *E. aerogenes* and one *E.coli* (Table 1). Clonality testing of *K. pneumoniae* and *E. cloacae* isolates showed clonally-distinct populations respectively. Ertapenem resistance was not mediated by carbapenemases or MBLs in all three species. Overall, 80% (12/15) of tested isolates had beta-lactamases (TEM-1, SHV-1, SHV-11, SHV-12, SHV-50, CTX-M-1) or AmpC genes of the DHA-type alone or in combinations.

## Discussion

Infection with carbapenem-resistant *Enterobacteriaceae* (CRE) is emerging as an important challenge in health-care settings [31]. While there are a number of studies relating to CRE in general, information pertaining to ERE is scarcer. Our study evaluated the potential risk factors and outcomes for the acquisition of ERE in hospitalized patients. We found that previous hospital stay and increased durations of prior fluoroquinolone therapy appeared to be associated with ERE acquisition. In addition, higher mortality rates and poorer clinical response rates were observed in ERE-infected patients.

Only two case-control studies investigating ERE have been published. Risk factors implicated in these studies included: ICU stay, exposure to invasive medical devices, and antibiotic exposure, specifically cephalosporin and carbapenem use [20], [21]. Our study, on the other hand, identified previous hospital stay as a significant independent predictor of ERE acquisition. This is not surprising as this factor has been widely acknowledged to be associated with the acquisition of several different types of antibiotic-resistant organisms such as multi-drug resistant *Acinetobacter baumanni*, and *Pseudomonas aeruginosa* [32], [33]. Previously hospitalized patients will have a higher likelihood of exposure to nosocomial organisms through horizontal transmission among patients. Furthermore, previous hospital stay could be a surrogate marker of prior antibiotic exposure.

Long durations of fluoroquinolone exposure were also associated with the acquisition of ERE in our study. This association offers biologic plausibility. Ertapenem resistance is commonly mediated by the production of AmpC/ESBL enzymes coupled with a deficiency in expression of the outer membrane proteins (OMPs) [7]–[10]. In our study, 80% of available isolates tested identified ESBL/AmpC as the prevalent mechanisms. There is a possibility that fluoroquinolone use has resulted in a selective pressure for these ESBL-producing *Enterobacteriaceae* in our institution. In this population of ESBL-producing *Enterobacteriaceae*, there could be an ertapenem-resistant subpopulation with deficiency in porin expression. This phenomenon has been demonstrated by Leavitt *et al.*, where 1–2% of ESBL-producing *Klebsiella pneumonia* in the study was found to be lacking in OMPK36 [10]. Further constant and cumulative antibiotic exposures, including ertapenem and the other carbapenems use, then exert a selective pressure for these ESBL-producing *Enterobacteriaceae* associated with porin loss, or facilitate the activation of mechanisms leading to porin loss, resulting in the predomination of ertapenem-resistant phenotypes.

Our study did highlight some key differences when compared to the other ERE and CRE risk factor studies whereby poor functional status; ICU stay; presence of biliary drains and invasive ventilation; prior beta-lactams, anti-pseudomonal penicillins and carbapenem exposure were identified as predictors [13]–[21]. These discrepancies in findings were not unexpected since the profiles of study organisms were largely different - some studies included both colonized patients and those with clinically-relevant infections; and sites and species of infecting organisms also varied. The resulting epidemiological characteristics and resistance mechanisms of study organisms were hence diverse. For instance, isolates from Israel had a mix of KPC- and ESBL-producers with porin loss [14], [16]; carbapenem-resistant strains from Greece were likely to be MBL-producers [34]; US isolates consisted of KPC-producers [35]–[37]; while our local isolates were predominantly ESBL-producers, likely with porin loss. This diversity in carbapenem resistance mechanisms may be associated with distinct clinical risk factors, in particular prior antibiotic exposure, explaining the differences between the studies.

Not surprisingly, we showed that there is a significant difference in in-hospital mortality and clinical response between the ERE and ESE group. However, ERE infection was not found to be a significant predictor of in-hospital mortality in the multivariate analysis, probably due to the confounding effect of severity of illness. A more extensive study with a larger sample size would have to be undertaken to better characterize the clinical outcomes.

Our study presented with several limitations. First, this is a retrospective study with a small sample size – only 29 cases in each case group were collected, limiting the power of the study. However, almost all adult hospitalized patients with an ERE during the study period were included. Molecular characterization of ERE was further limited to only 15 available isolates, hence results may not have been representative of the true distribution of resistance mechanisms locally. In addition, controls were not screened for *Enterobacteriaceae* by active surveillance, some patients with unrecognized *Enterobacteriaceae* colonization may be misclassified as non-infected control patients. Our study also did not seek to distinguish between colonization and infection with and ERE isolate, which could present very different epidemiological and clinical characteristics, and hence different risk factors.

As with many antimicrobial resistance epidemiological studies, selection bias and confounding are issues of concern. The use of controls without prior gram-negative bacillary infection, and with possible gram-positive infection only, may have introduced a selection bias towards potentially healthier controls. Furthermore, there is a concern that use of such a control group may not represent the true source population for the cases, and will not allow the differentiation between ERE-specific risk factors and risk factors relating to gram-negative bacilli infections in general. Missing information on antibiotic treatments and instrumentation at private healthcare providers prior admission, and the lack of electronic randomization procedures could have also contributed to selection bias. Finally, case and control patients were not matched due to difficulties in obtaining matched controls for two different case groups. This led to differences in certain variables such as length of stay prior to outcome of interest, severity of illness and age between the groups, all of which could potentially confound the interpretation of results. The differences in the median lengths of stay prior to outcome of interest between the ERE group and the two other groups, is a possible study limitation. However, it is also debatable, as a significantly longer hospitalization may be needed to acquire ERE. Future studies should be designed to control for these differences in length of stay.

In conclusion, we found that ERE infection was associated with previous hospital stay and increased duration of prior fluoroquinolone therapy, notwithstanding the above limitations. Patients with ERE infection experienced higher in-hospital mortality rates and poorer clinical response. ERE represent a major clinical and infection control challenge. Evidently, more active interventions and research in this area is required to curb the problem. In particular, the introduction of preventive measures such as antimicrobial stewardship to help reduce unnecessary fluoroquinolones use may help to attenuate the risk of ERE infections. Nosocomial transmission is a critical factor in the context of carbapenem-resistant *Enterobacteriaceae*, and risk of horizontal transmission during hospitalization can be alleviated by active surveillance, good infection control practices such as hand hygiene and cohorting measures. Further investigations on mechanisms of resistance and clonal spread, as well as a more detailed analysis of clinical outcomes are warranted for a better understanding of the current problem.
